# Urinary Sodium Excretion and Obesity Markers among Bangladeshi Adult Population: Pooled Data from Three Cohort Studies

**DOI:** 10.3390/nu14143000

**Published:** 2022-07-21

**Authors:** Musarrat J. Rahman, Sarker M. Parvez, Mahbubur Rahman, Feng J. He, Solveig A. Cunningham, K. M. Venkat Narayan, Jaynal Abedin, Abu Mohd Naser

**Affiliations:** 1 International Health, Johns Hopkins Bloomberg School of Public Health, Baltimore, MD 21205, USA; mrahma28@jhmi.edu; 2 Environmental Interventions Unit, Infectious Disease Division, International Centre for Diarrhoeal Disease Research, Bangladesh (icddr,b), Mohakhali, Dhaka 1212, Bangladesh; parvez@icddrb.org (S.M.P.); mahbubr@icddrb.org (M.R.); 3 Centre for Public Health and Policy, Wolfson Institute of Population Health, Barts and The London School of Medicine and Dentistry, Queen Mary University of London, London EC1M 6BQ, UK; f.he@qmul.ac.uk; 4 Emory Global Diabetes Research Center, Hubert Department of Global Health, Rollins School of Public Health, Emory University, Atlanta, GA 30322, USA; sargese@emory.edu (S.A.C.); knaraya@emory.edu (K.M.V.N.); 5 Data Science Institute, National University of Ireland Galway, H91 TK33 Galway, Ireland; joystatru@gmail.com; 6 Division of Epidemiology, Biostatistics and Environmental Health, School of Public Health, University of Memphis, Memphis, TN 38152, USA

**Keywords:** sodium intake, obesity, urine sodium, body mass index, waist circumference

## Abstract

We evaluated the relationship of urinary sodium excretion with a conditional mean, 10th and 90th percentiles of body mass index (BMI), and waist circumference among 10,034 person-visits of Bangladeshi population. We fitted linear mixed models with participant-level random intercept and restricted maximum likelihood estimation for conditional mean models; and quantile mixed-effect models with participant-level random intercept and Laplace estimation for 10th and 90th percentiles models. For each 100 mmol/24 h increase in urinary sodium excretion, participants had a 0.10 kg/m^2^ (95% CI: 0.00, 0.10) increase in the mean; a 0.39 kg/m^2^ (95% CI: 0.23, 0.54) increase in the 10th percentile; and a 0.59 kg/m^2^ (95% CI: 0.39, 0.78) increase in the 90th percentile of BMI. For each 100 mmol/24 h increase in urinary sodium excretion, participants had a 0.20 cm (95% CI: 0.10, 0.30) increase in mean; a 0.18 cm (95% CI: −0.03, 0.40) change in the 10th percentile; and a 0.23 cm (95% CI: 0.03, 0.43) increase in the 90th percentile of waist circumference. We found a modest association between urine sodium and conditional mean of BMI and waist circumference. The magnitude of associations between urine sodium and the 10th and 90th percentile BMI distributions were higher compared to the conditional mean models, suggesting high sodium intake could be more detrimental to underweight and obese participants.

## 1. Introduction

The prevalence of obesity worldwide has more than doubled in the past couple of decades [1,2]. The percentage increase in the number of global disability-adjusted life years (DALYs) attributable to a high body mass index (BMI) from 1990 to 2019 was 138% [3]. In 2016, around 39% of adults (≥18 years) were overweight, and 13% of adults were obese globally [4]. Obesity and being overweight are risk factors for cardiovascular diseases [5,6,7,8,9], diabetes mellitus [10,11], kidney diseases [12,13], and several types of cancers [14,15]. Several epidemiological studies have identified a link between sodium intake and multiple markers of obesity, such as body mass index (BMI), waist circumference, and body fat percentage [16,17,18,19]. Waist-to-hip ratio and waist-to-height ratio are additional obesity markers linked to metabolic risks [20,21,22,23].

The largest percentage increase in obesity-related deaths (158% increase) and DALYs (166% increase) from 1990 to 2017 occurred in South Asia [2]. South Asians are particularly vulnerable to cardiometabolic diseases [24,25,26] due to low muscle mass, visceral fat accumulation, and high abdominal mass [27]. However, links between sodium intake and obesity markers have not been explored among South Asians, particularly among the Bangladeshi population [19]. High sodium consumption is common among South Asians, where the source of sodium is the use of high levels of salt during the cooking process and the use of table salt during meals [28,29]. WHO recommends reducing sodium intake to <2 gms/day for adults [30]. This is equivalent to <87 mmol of sodium/day or 5 gms of salt/day in adults. Studies from Bangladesh have shown higher sodium intake. One study found mean urinary sodium excretion to be 115 mmol/day, equivalent to 6.8 gms of salt/day, among healthy adults [31]. Two more studies on pregnant women in southwest coastal Bangladesh found mean urinary sodium excretion to be 3.4 g/day (8.5 g salt), or 170 mmol/day (10 g salt) [32] and 180 mmol/day (10.6 gms salt) for those who particularly drank tube well water [33]. Other studies have estimated sodium intake to be as high as 16 g of salt/day in the dry season for those who consume brackish drinking water in coastal Bangladesh [34,35]. In comparison, sodium consumption has been found to be 3.8 gms/day among US adults [36], and 3.04 gms/day among UK adults [37].

One reason for the lack of evidence of the sodium–obesity link among the Bangladeshi population is the lack of reliable longitudinal data on sodium intake where sodium intake was measured in 24 h urine samples. Around 93% of sodium is excreted via 24 h urine, making it a gold-standard marker for daily sodium intake [38]. Furthermore, most epidemiological studies implemented globally investigated the relationship between sodium intake and the conditional mean of obesity markers. Nevertheless, the public health significance of the sodium–intake–obesity relationship can differ for populations in extreme percentiles of obesity markers’ distribution. For instance, several studies highlighted that both underweight and overweight individuals have high risks for metabolic diseases [39,40]. Public health communities may be more interested in investigating the sodium–intake–obesity relationship among people with underweight or extreme obesity. Currently, limited scientific knowledge exists on the sodium–intake–obesity relationship for underweight or overweight Bangladeshi populations. The overarching objective of this paper is to characterize the relationships of sodium intake with the conditional mean, 10th percentile, and 90th percentile distribution of several obesity markers among the Bangladeshi population.

## 2. Materials and Methods

We pooled 10,034 person-visits data of 1850 persons of ≥20 years from three longitudinal studies conducted in communities in southwest coastal and central Bangladesh [41]. A detailed description of how study participants were selected is provided elsewhere [41] (see Supplementary Figure S2). Populations in southwest coastal Bangladesh have a high sodium intake through drinking water during the dry season due to saltwater intrusion from the sea [42]. The first study followed 383 participants from 166 households from 4 villages in two visits (from May 2016 to August 2016) when the sodium content in drinking water was low [43]. The second study was a population-based stepped-wedged cluster randomized trial that followed 1190 participants from 16 villages in southwest coastal Bangladesh in 5 monthly visits during the dry season (from December 2016 and April 2017) when sodium content of the drinking water was high [44]. The third study followed 293 participants from coastal and 277 from non-coastal regions for 7 visits longitudinally through the wet and dry seasons (from August 2018 to April 2019) [45]. The 293 participants from the coastal area in the third study were taken from the second study, and so we analyzed data from a total of 1850 unique participants. Each participant was visited an average of 5 times, and a minimum of 2 and maximum of 12 times, capturing seasonal variation in sodium intake in our study population.

### 2.1. 24 h Urine Collection and Sodium Measurement

To collect a 24 h urine sample, research assistants provided all participants with a 4-L plastic container and a mug to transfer the voided urine to the container. Research assistants instructed participants to begin collecting from their second void of the first day, and then transfer all other voids for the day up until the first void for the subsequent morning [46]. The total volume of the 24 h urine was recorded. Then, after stirring the 4-L container, a 15 mL sample was extracted and transported to a field-based laboratory at 2 to 8 °C for same-day processing, aliquoting, and analysis in the laboratory. We used the direct ion-selective method for 24 h urinary sodium measurements, which is commonly used in clinical biochemistry laboratories [47] in a semi-automatic electrolyte analyzer (Biolyte2000, Bio-care Corporation, Taoyuan, Taiwan; coefficient of variation: 5%).

### 2.2. Obesity Marker Measurement

Participants’ body weight was measured to the nearest 0.1 kg in each visit using a Body Composition Monitor (HBF-214^®^, Omron, Kyoto, Japan) on a flat and solid surface. Trained data collectors ensured that the participants were wearing light clothing at the time of measurement. An average of 3 measurements was used for the weight analyses. Participants’ height was measured once, usually during the first visit of each study, using a ShorrBoard^®^ (Weigh and Measure, LLC, Olney, MD, USA) Adult Portable Height–Length Measuring Board to the nearest 0.1 cm. Trained data collectors ensured that the participants stood on a flat surface with feet parallel and pointing forwards, arms hanging freely from the sides, and head positioned straight with eyes looking forward.

Waist and hip circumferences were measured to the nearest 0.1 cm using a measuring tape. Research staff positioned the measuring tape around the waist parallel to the floor, midway between the upper-most border of the iliac crest and the lower border of the coastal rib cage. For hip circumference, the tape was positioned parallel to the convex part of the hip, at the point that yielded the maximum circumference while the participant was standing. The research staff took measurements at the end of the exhalation. The mean of three measurements was used for analyses.

We additionally measured body fat percentage and visceral fat percentage data from 570 participants from the third study. The Body Composition Monitor (HBF-214) also provided readings on body fat percentage and visceral fat level using the bioelectrical impedance method, which is considered well-adapted for low-resources settings for relatively simple use [48].

### 2.3. Covariates

We collected data on demographic variables and relevant cardiovascular disease risk factors during the baseline visit for each study. These included age (continuous), sex (male, female), physical exercise (sedentary, moderate-intensity activity and vigorous-intensity activity based on WHO STEPS [49]), alcohol consumption (yes, no), hours of sleep (<6 h, ≥6 to <9 h, ≥9 h), smoking status (never, former, current), religion (Hindu, Muslim), location (coastal, non-coastal), season (dry, wet), and household assets quintile. We also collected participant-reported information about their hypertension (yes, no), diabetes (yes, no), kidney disease (yes, no), heart disease (yes, no), and stroke status (yes, no). The dry season data were all the data collected from December to April, and data from May to October were considered as wet season data.

### 2.4. Statistical Analyses

We reported the mean and 95% confidence interval (CI) for continuous variables and percentage categorical variables. We created a wealth index (asset quintiles) using polychoric principal component analysis (PCA) on the household asset ownership variables: refrigerator, television, mobile phone, motorcycle, bicycle, sewing machine, chair, table, wristwatch, wardrobe, wooden cot, motor pump, rice husking machine, motorized rickshaw, car, and access to electricity [50,51,52]. The first principal component score was then converted into five quintiles with the lowest quintile representing the poorest category. To understand whether participants’ characteristics vary across sodium excretion group, we summarized participants’ characteristics across tertiles of 24 h urine sodium excretion. We created tertiles of 24 h urinary sodium excretion—tertile 1 (below 118.63 mmol/24 h), tertile 2 (from 118.64 to 173.74 mmol/24 h), and tertile 3 (above 173.80 mmol/24 h). Further analyses were conducted with 24 h urinary sodium as a continuous variable.

To explore the relationship between 24 h urinary sodium excretion and obesity markers, we initially visually examined the unadjusted relationship between urine sodium excretion and obesity markers using cubic spline for urine sodium with three cut points at 10, 50, and 90th percentile distributions [53]. Since we did not find any non-linear association on visual inspection (Supplementary Figures S1 and S2), we then fitted linear mixed effect models to evaluate the relationship between 24 h urine sodium excretion and each of the obesity markers. We used participant-level random intercept and restricted maximum likelihood for model estimation to adjust clustering of data due to repeated visits.

We sequentially adjusted for covariates. Model 1 reported unadjusted estimates. Model 2 reported estimates adjusted for age, sex, and weight or height, depending on obesity markers. For the waist-to-height ratio, in Model 2, we included weight, in addition to age and sex. Likewise, we included both height and weight for waist-to-hip ratio, waist circumference, total body fat, and visceral fat outcomes, in addition to age and sex in Model 2. Height and weight were used to define BMI, so we did not adjust for these in the Model 2 when BMI was an outcome.

Model 3 additionally adjusted for physical exercise, sleep, and smoking. Model 4 additionally adjusted for religion, location (coastal vs. non-coastal), asset quintiles, and season (dry or wet); Model 5 adjusted for the presence of self-reported comorbid conditions such as hypertension, diabetes, any kidney disease, heart disease, and stroke. Age, height, weight, BMI, waist circumference, waist-to-hip ratio, and waist-to-height ratio were continuous variables and all other covariates were categorical variables. We checked for multicollinearity by calculating the variance inflation factor (VIF).

To evaluate the sodium–intake–obesity relationship among underweight or overweight Bangladeshi populations, we used a linear quantile mixed effect regression [54] with participant-level random intercept and the Laplace estimation method. We represented 10th percentile distributions of obesity markers as underweight and 90th percentile distributions as the overweight population. Only full adjusted models were implemented for the quantile regression.

### 2.5. Reproducibility and Sensitivity Analysis

In the data preparation step, we included 8820 observations (1440 participants) after dropping 1211 observations that contained implausible and missing values in either the outcome variable or any one of the covariates under consideration. There was no specific pattern found in the dropped rows of the data from which we can safely assume that the data were missing at random and it did not affect any of our results.

To ensure reproducibility of the results reported in this paper, we have used R programming language and written detailed R scripts [55]. To further validate and ensure reproducibility, one of the co-authors independently conducted the analysis and verified the results. The R scripts for the entire analysis will be made available after acceptance in the Journal for publication for the readers upon request to the corresponding author.

One limitation of using 24 h urine sodium as a proxy of sodium intake is that 24 h urine collection can be biased due to under or over collection. Under collection is common since participants may forget to transfer void to 24 h urine storage containers. To avoid bias due to under collection, in a sensitivity analysis, we used a creatinine-based method to exclude under-collected samples. We excluded samples if the creatinine index was <0.7 [56].

### 2.6. Ethics

Written informed consent was obtained from all participants. The study protocols were approved by the Ethical Review Committee of the International Centre for Diarrhoeal Disease Research, Bangladesh (icddr,b) (PR-15096, approved 10 March 2016).

## 3. Results

The pooled cohort data consisted of 1833 persons (8820 person-visits) after adjusting for missing values. The mean age of respondents was 42.37 years (95% CI: 41.70, 43.04) and 729 (39.77%) were male (Table 1). The mean BMI of respondents was 22.24 kg/m^2^ (95% CI: 22.04, 22.44) and the mean waist circumference was 80.04 cm (95% CI: 79.56, 80.52) (Table 1). The mean 24 h urinary sodium excretion was 153.89 (95% CI: 150.71, 157.07) mmol/24 h. Participants in the highest sodium excretion tertile had higher weight, height, BMI, and waist circumferences compared to participants in the lowest sodium excretion tertile. Twenty-four-hour urinary sodium excretion was higher among participants in the highest asset quintile relative to the lowest asset quintile. Twenty-four-hour urinary sodium excretion was also higher among participants with no cardiovascular diseases relative to participants with cardiovascular diseases. Coastal populations had a much higher 24 h urinary sodium excretion relative to non-coastal populations.

### 3.1. Urine Sodium and Conditional Mean of Obesity Markers

A 100 mmol/24 h increase in urinary sodium excretion was associated with a 0.20 cm (95% CI: 0.10, 0.30) increase in waist circumference, and a 0.10 kg/m^2^ (95% CI: 0.00, 0.10) increase in BMI in the full-adjusted-multivariable models (Table 2 and Table 3). For each 100 mmol/24 h increase in urinary sodium excretion, participants had a 0.00 kg/m^2^ (95% CI: −0.001, 0.002) change in waist-to-hip ratio, and a 0.001 (95% CI: 0.001, 0.002) increase in waist-to-height ratio in the full-adjusted-multivariable models (Table 4 and Table 5).

For each 100 mmol/24 h increase in urinary sodium excretion, participants had a 0.10 (95% CI: −0.20, 0.30) percent change in body fat (Table 6), and −0.0 (95% CI: −0.10, 0.10) percent change in visceral fat (Table 7).

### 3.2. Urine Sodium and 90th Percentile Distribution of Obesity Markers

In the full-multivariable-adjusted model, a 100 mmol/24 h increase in urinary sodium was associated with a 0.23 cm (95% CI: 0.03, 0.43) increase in waist circumference; a 0.59 kg/m^2^ (95% CI: 0.39, 0.78) increase in BMI (Table 8); and a 0.003 (95% CI: 0.00, 0.006) increase in waist-to-hip ratio. For each 100 mmol/24 h increase in urinary sodium, participants had a 0.001 (95% CI: −0.003, 0.001) change in waist-to-height ratio (Table 8); a 0.19 (95% CI: −0.01, 0.39) change in body fat percentage (Table 9); and a −0.04 (95% CI: −0.12, 0.04) change in visceral fat (Table 9).

### 3.3. Urine Sodium and 10th Percentile Distribution of Obesity Markers

In the full-multivariable-adjusted model, for each 100 mmol/24 h increase in urinary sodium, participants had a 0.18 cm (95% CI: −0.03, 0.40) change in waist circumference; a 0.39 kg/m^2^ (95% CI: 0.23, 0.54) increase in BMI (Table 10); a 0.003 (95% CI: 0.000, 0.005) change in waist-tohip ratio; a −0.001 (95% CI: 0.003, 0.001) change in waist-to-height ratio; a 0.19 (95% CI: −0.01, 0.40) change in the total body fat percentage (Table 11); a −0.04 (95% CI: −0.12, 0.04) change in visceral fat (Table 11).

The sensitivity analyses provided almost similar estimates when we restricted the analyses among complete 24 h urine samples based on creatinine index ≥0.7 (Table 12). We did not find multicollinearity using VIF: none of the covariates had VIF over the critical threshold of 2.5.

## 4. Discussion

We found that higher sodium intake, as measured by 24 h urinary sodium excretion, had a modest positive association with waist circumference and BMI after adjustment of all the potential confounders, but no association with waist-to-hip and the waist-to-height ratios. Compared to the conditional mean of BMI, the associations of 24 h urinary sodium excretion and BMI was almost six times higher for the 90th percentile distribution of BMI, and almost four times higher for the 10th percentile distribution of BMI.

The mean urinary excretion level of 153.89 mmol/day found in our study from southwest coastal Bangladesh was higher than the estimate of 115 mmol/day found from southeast coastal healthy adults population in Bangladesh [31], and it was lower than the estimates from southwest coastal pregnant women in Bangladesh, which were 170 mmol/day [32] and 180 mmol/day [33]. Nonetheless, a mean of 153.89 mmol/day is still much higher than the recommended level of <87 mmol of sodium per day by WHO.

Studies on rats have shown a possible biological mechanism behind the sodium intake and obesity link. Sodium intake can enhance the lipogenic enzymatic activities by increasing plasma leptin production that induce adipocyte hypertrophy and excessive fat accumulation [57,58]. A high sodium-containing diet activates the endogenous fructose production in liver that then induces leptin resistance and obesity [59]. Another possible explanation for the sodium intake and obesity link is an indirect one—that high sodium intake is associated with overall caloric intake and that influences obesity. However, multiple past studies have established the association between sodium intake and obesity after controlling for energy intake [17,23,37,60,61] and consumption of sugary beverages [60,61,62,63]. This means that there is some sort of a direct biological mechanism at play whereby salt intake alters body fat metabolism. One study controlled for total body fat percentage in order to exclude sodium-related water retention as a possible reason for sodium intake and body weight [64]. They found a weak association between dietary sodium intake and total body fat percentage, strengthening the possibility of a direct biological link between sodium intake and obesity.

Our study findings are consistent with several other epidemiological studies highlighting a positive association between sodium consumption, waist circumference, and BMI [19,60,61,65,66]. A study among 16,000 diverse US Hispanics/Latinos found that each 500 mg (approximately 21.75 mmol) increment of daily dietary sodium was significantly associated with a 0.07 kg/m^2^ higher BMI (95% CI: 0.00, 0.15) and 0.18 cm larger waist circumference (95% CI: 0.00, 0.36) [67]. National Health and Nutrition Examination survey (NHANES) data suggest that a 1000 mg/24 h (approximately 42.5 mmol) higher sodium excretion was significantly associated with a 3.8 kg/m^2^ (95% CI: 2.8, 4.8) higher BMI and a 9.2 (95% CI: 6.9, 11.5) cms greater waist circumference [61]. Another study using the NHANES data found that for each 1000 mg/24 h increase in sodium intake, BMI was 1.03 kg/m^2^ higher (95% CI: 0.70, 1.35) and waist circumference was 2.15 cms (95% CI: 1.41, 2.90) higher [68]. A study of Australian school children found that an additional 17 mmol/24 h of sodium was associated with (approximately 390 mg/24 h or 1 g salt/24 h of sodium) 0.1 higher BMI z-score (95% CI 0.07, 0.13) [23]. In all these studies, the strength of association found for the influence of sodium intake on obesity markers is much higher than the strength of association we found for our study [23].

The association between sodium intake and various obesity markers can differ across different groups of people based on factors such as age, ethnicity, and gender. A meta-analysis of 11 cohort and 21 cross-sectional studies found high heterogeneity in the association between sodium consumption and obesity markers across subgroups such as sex and sodium assessment tools [19]. For example, subgroup analyses showed a significant increment in waist circumference in females, but the association was not significant for males. Furthermore, a multi-country analysis found that sodium intake of 1 g/24 h (1000 mg/24 h; approximate 42.5 mmol salt/24 h) was associated with a higher BMI by 0.28 (95% CI: 0.23, 0.34) in Japan, 0.10 (95% CI: 0.05, 0.14) in China, 0.42 (95% CI: 0.27, 0.56) in the United Kingdom and 0.52 (95% CI: 0.45, 0.59) in the United States [69]. The strength of associations found in relation to Chinese and Japanese populations is somewhat similar to the strength of association we found in our study for BMI.

Our study on Bangladeshi populations did not find an association of urine sodium excretion with waist-to-hip and waist-to-height ratios. A cross-sectional study of 640 healthy adults from South Korea established an association of 24 h urinary sodium with waist-to-hip ratio and waist-to-height ratio [22]. Waist-to-hip ratio and waist-to-height ratios can provide better reflection of inflammation and cardiometabolic risks relative to BMI [70,71,72]. The predictive power of each of the different obesity markers seem to vary based on ethnicity, age, and sex [73] and so, it is likely that the waist-to-hip and the waist-to-height ratio indicators are not sensitive enough to observe an association regarding sodium excretion in Bangladeshi populations.

Even though a prior study found a weak association between dietary sodium intake and total body fat percentage [64], we did not find any significant association of urine sodium with total body and visceral fat percentage in the fully adjusted models. One possible explanation for no association is that the models were adjusted for weight--a potential confounder in our analyses. Moreover, the analysis for body and visceral fat data as outcomes was conducted using a small subset of the total study population for whom this data was available. The exposure to sodium intake through drinking water was low for this subset of the population.

Our study had some strengths. Firstly, we had a large sample size for a variety of obesity markers collected in all three studies that provided enough power for analysis. We used random intercepts to control for unmeasured individual-level non-time-varying confounders. Using data from geographical locations in coastal (where drinking water sources are susceptible to saline intrusion from seawater) and non-coastal areas, we were able to obtain a wide variation of sodium consumption for our analysis. Secondly, our study included multiple markers of obesity. Past studies have relied on BMI as the primary measure of obesity. However, BMI is an indicator of general obesity and does not consider body fat distribution. Waist circumference, waist-to-hip ratio, and waist-to-height ratio are measures of abdominal obesity which consider body fat distribution and, in some cases, are better predictors of metabolic complications [74,75,76,77,78] and cardiovascular outcomes [79,80,81,82,83,84,85] relative to BMI. Furthermore, BMI is unable to distinguish between lean and fat mass which can lead to misclassification [86]. For example, South Asians have higher levels of body fat percentage and visceral fat relative to Caucasians at any given BMI level, and this leads to a higher prevalence of metabolic diseases [87,88,89]. Particularly, abdominal visceral fat, as opposed to subcutaneous fat, is linked to multiple adverse metabolic outcomes of obesity [90,91]. As mentioned earlier, the predictive power of each of these obesity markers seems to vary with ethnicity, age, and sex [73], and so exploring which obesity markers are particularly affected by sodium intake in the Bangladeshi population is crucial. Thirdly, an additional advantage of the current analysis is our quantile regression analyses that identified a higher magnitude of association between 24 h urinary sodium with high and low BMI distributions than the mean models. Studies typically explore the association between sodium intake and mean obesity markers. We found a stronger magnitude of association between sodium intake and obesity markers for high and low BMI groups. Prior research suggests that both underweight and overweight are associated with an increased risk of all-cause mortality relative to normal BMI [92]. The relationship between BMI and mortality is U-shaped or J-shaped. South Asians with low BMI usually suffer from undernutrition, have low muscle mass, and a high risk of developing insulin resistance [93]. Due to undernutrition since childhood, their body systems are not well developed, making them more vulnerable to dietary risk factors [94], including high sodium intake. In contrast, the presence of comorbid conditions and other risk factors among high BMI individuals can modify the effect of sodium intake and obesity markers.

Our study also had some limitations. Twenty-four-hour urinary excretion is the gold standard for estimating daily sodium intake. However, collection can be susceptible to over-collection and under-collection [56]. In order to estimate for completeness of urine collection, para-aminobenzoic acid is recommended for use in a subsample [95]. However, the current study was unable to do this. Therefore, our estimates may be influenced by over-or under-collection. However, we found similar results when we restricted our analyses to complete 24 h urine samples derived by the indirect method of creatinine index. A second limitation is that temperature and physical activities can affect the rate of sweating and sodium excretion [96]. Men in rural areas especially are involved in various physical labor in the hot humid climate, which makes them sweat. It is likely that a fair amount of sodium excretion occurs as a result of sweating, making the urinary sodium data an underestimate. However, sodium excretion via sweat is reduced in acclimatized individuals within a couple of weeks [97,98], and so it is also likely that our urinary estimates are underestimates by only a small margin. A third limitation is that while weight and height can be measured precisely, waist and hip circumferences measurements can have strong between-observer differences [99]. Since the current study had a large sample size, we had to employ multiple data collectors. However, we tried to minimize the possibility of error through strong training and taking the average of three measurements. We could not control for calorie intake in the association between sodium intake and obesity because we did not collect energy intake data. While this is a limitation in the current study, as mentioned earlier, past studies [17,23,37,60,61,62,63] that have controlled for calorie intake, energy intake, and sugary beverages, also found a link between sodium intake and obesity markers. Finally, we were unable to adjust for lipid abnormalities in our analyses since we did not collect lipid data in all visits for this population-based study. High blood cholesterol can lead to renal vasoconstriction as a result of decreased nitric oxide production and subsequent anti-natriuretic responses [100]. High blood cholesterol is an important risk factor for hypertension. One of the mechanisms of cholesterol-induced high blood pressure is increased sodium and fluid reabsorption by altering the Na+ channel in renal epithelium [101].

## 5. Conclusions

Our study findings suggest that sodium intake is related to obesity markers. The next step would be to explore the link between sodium intake and obesity-related markers in blood and urine through rigorous epidemiological studies. Given the rising obesity epidemic and high sodium among South Asians, such research would provide important clues to identify interventions to lower cardiometabolic diseases. 

## Figures and Tables

**Table 1:** Characteristics of participants.

Characteristics	Participants (N: 1833)	Tertile 1 <118.63 mmol/24 h	Tertile 2 118.64–173.74 mmol/24 h	Tertile 3 >173.80 mmol/24 h and above
Age in years, mean (95% CI *)	42.37 (41.70, 43.04)	43.64 (42.35, 44.93)	41.72 (40.62, 42.83)	41.75 (40.67, 42.83)
Male sex, % (*n*)	39.77 (729)	39.93 (244)	40.26 (246)	39.12 (239)
Weight in kg, mean (95% CI)	53.95 (53.47, 54.44)	51.66 (50.85, 52.47)	53.61 (52.84, 54.39)	56.58 (55.70, 57.46)
Height in kg, mean (95% CI)	155.85 (155.45, 156.24)	154.73 (154.02, 155.44)	155.89 (155.23, 156.55)	156.91 (156.25, 157.57)
Body mass index, mean (95% CI)	22.24 (22.04, 22.44)	21.71(21.29, 22.12)	22.05 (21.77, 22.34)	22.97 (22.66, 23.27)
Waist circumference, mean (95% CI)	80.04 (79.56, 80.52)	78.31 (77.46, 79.16)	79.64 (78.86, 80.42)	82.17 (81.33, 83.01)
Body fat percentage	27.94 (26.99, 28.89)	27.02 (25.60, 28.44)	28.12 (26.23, 30.01)	30.23 (28.64, 31.83)
Visceral fat	6.31 (5.77, 6.84)	5.97 (5.17, 6.76)	6.89 (5.86, 7.92)	6.43 (5.41, 7.45)
24 h urinary sodium in mmol/24 h, mean (95% CI)	153.89 (150.71, 157.07)	85.66 (83.66, 87.65)	144.81 (143.62, 145.97)	231.22 (226.93, 235.51)
Volume of 24 h urine in L, mean (95% CI)	2.16 (2.11, 2.21)	1.58 (1.53, 1.64)	2.29 (2.22, 2.36)	2.60 (2.52, 2.69)
WHO work-related physical activity ** % (*n*)				
Sedentary	30.39 (557)	30.28 (185)	33.06 (202)	27.82 (170)
Moderate	47.52 (871)	51.55 (315)	45.17 (276)	45.83 (280)
Vigorous	22.09 (405)	18.17 (111)	21.77 (133)	26.35 (161)
Hours of sleep, % (*n*)				
<6 h	18.49 (339)	21.60 (132)	16.37 (100)	17.51 (107)
≥6 to <9 h	70.21 (1287)	66.94 (409)	73.32 (448)	70.38 (430)
≥9 h	11.29 (207)	11.46 (70)	10.31 (63)	12.11 (74)
Smoking categories, % (*n*)				
Never	55.26 (1013)	56.63 (346)	52.70 (322)	56.46 (345)
Former	11.02 (202)	13.75 (84)	9.82 (60)	9.49 (58)
Current	33.72 (618)	29.62 (181)	37.48 (229)	34.04 (208)
Muslims, % (*n*)	54.56 (1000)	65.63 (401)	50.08 (306)	47.95 (293)
Location, % (*n*)				
Coastal	85.11 (1560)	76.43 (467)	89.53 (547)	89.36 (546)
Non-coastal	14.89 (273)	23.57 (144)	10.47 (64)	10.64 (65)
Alcohol, % (*n*)				
Yes	3.49(64)	4.09 (25)	3.60 (22)	2.78 (17)
No	96.51 (1769)	95.91(586)	96.60 (589)	97.22 (594)
Asset quantile, % (*n*)				
Lowest	17.04 (312)	16.39 (100)	16.37 (100)	18.36 (112)
Second	18.24 (334)	18.69 (114)	15.88 (97)	20.16 (123)
Third	19.88 (364)	20.66 (126)	20.62 (126)	18.36 (112)
Fourth	20.97 (384)	21.31 (130)	21.28 (130)	20.33 (124)
Highest	23.87 (437)	22.95 (140)	25.86 (158)	22.79 (139)
Season, % (*n*)				
Wet	18.5 (1865)			
Dry	81.5 (8211)			
Hypertension, % (*n*)				
Yes	15.22 (279)	14.73 (90)	15.71 (96)	15.22 (93)
No	84.29 (1545)	84.62 (517)	83.80 (512)	84.45 (516)
Do not know	0.49 (9)	0.65 (4)	0.49 (3)	0.33 (2)
Diabetes, % (*n*)	*n* = 1810	*n* = 602		*n* = 606
Yes	5.36 (97)	5.81 (35)	4.15 (25)	6.11 (37)
No	93.92 (1700)	93.52 (563)	94.68 (570)	93.56 (567)
Do not know	0.72 (13)	0.66 (4)	1.16 (7)	0.33 (2)
Any kidney disease, % (*n*)	*n* = 1830	*n* = 610	*n* = 610	*n* = 610
Yes	2.30 (42)	2.13 (13)	2.30 (14)	2.46 (15)
No	96.12 (1759)	95.57 (583)	96.39 (588)	96.39 (588)
Do not know	1.58 (29)	2.30 (14)	1.31 (8)	1.15 (7)
Any heart disease, % (*n*)	*n* = 1801	*n* = 596	*n* = 603	*n* = 602
Yes	4.44 (80)	4.19 (25)	4.31 (26)	4.82 (29)
No	94.78 (1707)	94.63 (564)	94.86 (572)	94.85 (571)
Do not know	0.78 (14)	1.17 (7)	0.83 (5)	0.33 (2)
Stroke, % (*n*)	*n* = 1801	*n* = 596	*n* = 603	*n* = 602
Yes	2.67 (48)	2.52 (15)	3.32 (20)	2.16 (13)
No	97.22 (1751)	97.32 (580)	96.68 (583)	97.67 (588)
Do not know	0.11 (2)	0.17 (1)	-	0.17 (1)

* Tertile 1: 24 h urinary sodium from 8.87–118.63 mmol/24 h; Tertile 2: 24 h urinary sodium from 118.64 to 173.74 mmol/24 h; Tertile 3: 24 h urinary sodium from 173.80–1843.40 mmol/24 h; CI: confidence interval; ** Sedentary behavior was defined as sitting or reclining at work, at home, getting to and from places, or with friends, including time spent sitting at a desk, sitting with friends, travelling within a car, bus, or train, reading, playing cards, or watching television but not time spent sleeping. Moderate-intensity activities were defined as activities that cause small increases in breathing or heartrate such as brisk walking (or carrying light loads) for at least 10 min continuously. Vigorous-intensity activities were defined as activities that cause large increases in breathing or heart rate, like carrying or lifting heavy loads, digging or construction work, for at least 10 min continuously. Hours of sleep were categorized as <6 h, ≥6 to <9 h, and ≥9 h. Smoking status was categorized as never, former, and current.

**Table 2:** Association between 24 h urinary sodium and mean waist circumference (cms).

	Model 1			Model 2			Model 3			Model 4			Model 5		
Predictors	Estimates	95% CI	*p*-Value	Estimates	95% CI	*p*-Value	Estimates	95% CI	*p*-Value	Estimates	95% CI	*p*-Value	Estimates	95% CI	*p*-Value
Intercept	78.9	78.3–79.4	<0.001	77.9	72.7–83.1	<0.001	77.9	72.7–83.1	<0.001	77.2	72.2–82.3	<0.001	77.2	72.2–82.3	<0.001
Sodium (100 mmol)	0.2	0.1–0.3	<0.001	0.2	0.0–0.3	<0.001	0.2	0.1–0.3	<0.001	0.2	0.1–0.3	<0.001	0.2	0.1–0.3	<0.001
Age (Years)				0.1	0.1–0.1	<0.001	0.1	0.1–0.1	<0.001	0.1	0.1–0.1	<0.001	0.1	0.1–0.1	<0.001
Sex (Male)				0.4	−0.2–0.9	0.17	0.4	−0.1–1.0	0.14	0.3	−0.3–0.8	0.33	0.3	−0.3–0.8	0.33
Height (cms)				−0.4	−0.4–−0.3	<0.001	−0.4	−0.4–−0.3	<0.001	−0.3	−0.4–−0.3	<0.001	−0.3	−0.4–−0.3	<0.001
Weight (kg)				1.0	1.0–1.0	<0.001	1.0	1.0–1.0	<0.001	1.0	1.0–1.0	<0.001	1.0	1.0–1.0	<0.001
Physical Exercise (Yes)							−0.2	−0.3–−0.1	<0.001	−0.2	−0.3–−0.1	<0.001	−0.2	−0.3–−0.1	<0.001
Sleep (Yes)							0.0	−0.2–0.2	0.76	0.0	−0.2–0.2	0.76	0.0	−0.2–0.2	0.77
Smoker (Yes)							0.1	−0.1–0.2	0.44	0.2	0.1–0.3	0.01	0.2	0.1–0.3	0.01
Drink Alcohol (Yes)							−0.4	−1.2–0.5	0.38	−0.3	−1.1–0.5	0.49	−0.3	−1.1–0.5	0.48
Religion (Islam)										0.3	−0.1–0.8	0.14	0.3	−0.1–0.8	0.14
Location (Coastal)										−2.3	−2.8–−1.7	<0.001	−2.3	−2.8–−1.7	<0.001
Asset Quintile										0.0	−0.1–0.2	0.90	0.0	−0.1–0.2	0.90
Season (Wet)										0.5	0.3–0.7	<0.001	0.5	0.3–0.7	<0.001
Hypertension													−0.0	−0.0–0.0	0.99
Diabetes													0.0	−0.0–0.0	0.28
Any kidney disease													−0.0	−0.0–0.0	0.20
Any heart disease													−0.0	−0.0–0.0	0.21
Stroke													−0.0	−0.0–0.0	0.82
Random Effects															
σ^2^	9.16			7.45			7.44			7.41			7.41		
τ_00_	97.90 _partID_			14.84 _partID_			14.83 _partID_			13.84 _partID_			13.84 _partID_		
ICC	0.91			0.67			0.67			0.65			0.65		
N	1440 _partID_			1440 _partID_			1440 _partID_			1440 _partID_			1440 _partID_		
Observations	8820			8820			8820			8820			8820		
Marginal R^2^/Conditional R^2^	0.000/0.915			0.797/0.932			0.797/0.932			0.805/0.932			0.806/0.932		

Model 1 reports unadjusted estimates; Model 2 has been adjusted for age, sex, height, and weight; Model 3 has been adjusted for age, sex, height, weight, physical exercise, sleep, and smoking; Model 4 has been adjusted for age, sex, height, weight, physical exercise, sleep, smoking, religion, location, asset quintiles, and season; Model 5 has been adjusted for age, sex, height, weight, physical exercise, sleep, smoking, religion, location, asset quintiles, season, hypertension, diabetes, any kidney disease, heart disease, and stroke; σ^2^: Population variance; τ_00_: Random intercept variance; ICC: Intraclass correlation coefficient.

**Table 3:** Association between 24 h urinary sodium and mean BMI (kg/m^2^).

	Model 1			Model 2			Model 3			Model 4			Model 5		
Predictors	Estimates	95% CI	*p*-Value	Estimates	95% CI	*p*-Value	Estimates	95% CI	*p*-Value	Estimates	95% CI	*p*-Value	Estimates	95% CI	*p*-Value
Intercept	22.2	22.0–22.4	<0.001	22.8	22.3–23.3	<0.001	23.3	22.7–23.8	<0.001	22.5	21.8–23.3	<0.001	22.5	21.8–23.3	<0.001
Sodium (100 mmol)	0.1	0.0–0.1	<0.001	0.1	0.0–0.1	<0.001	0.1	0.0–0.1	<0.001	0.1	0.0–0.1	<0.001	0.1	0.0–0.1	<0.001
Age (Years)				−0.0	−0.0−0.0	<0.001	−0.0	−0.0–−0.0	<0.001	−0.0	−0.0–−0.0	<0.001	−0.0	−0.0–−0.0	<0.001
Sex (Male)				0.0	−0.2–0.3	0.79	0.1	−0.2–0.3	0.49	0.1	−0.2–0.3	0.50	0.1	−0.2–0.3	0.50
Physical Exercise (Yes)							−0.1	−0.1–−0.0	<0.001	−0.1	−0.1–−0.0	<0.001	−0.1	−0.1–−0.0	<0.001
Sleep (Yes)							0.0	−0.0–0.1	0.65	0.0	−0.0–0.1	0.65	0.0	−0.0–0.1	0.69
Smoker (Yes)							−0.2	−0.2–−0.1	<0.001	−0.2	−0.2–−0.1	<0.001	−0.2	−0.2–−0.1	<0.001
Drink Alcohol (Yes)							0.2	−0.1–0.4	0.28	0.2	−0.1–0.4	0.24	0.2	−0.1–0.4	0.24
Religion (Islam)										0.4	−0.0–0.8	0.06	0.4	−0.0–0.8	0.06
Location (Coastal)										−0.2	−0.7–0.3	0.40	−0.2	−0.7–0.3	0.39
Asset Quintile										0.2	0.1–0.3	<0.001	0.2	0.1–0.3	<0.001
Season (Wet)										0.0	−0.0–0.1	0.39	0.0	−0.0–0.1	0.33
Hypertension													−0.0	−0.0–0.0	0.18
Diabetes													0.0	−0.0–0.0	0.27
Any kidney disease													−0.0	−0.0–0.0	0.21
Any heart disease													0.0	−0.0–0.0	0.20
Stroke													0.0	−0.0–0.0	0.97
Random Effects															
σ^2^	0.52			0.52			0.52			0.52			0.52		
τ_00_	13.69 _partID_			13.52 _partID_			13.32 _partID_			13.02 _partID_			13.01 _partID_		
ICC	0.96			0.96			0.96			0.96			0.96		
N	1440 _partID_			1440 _partID_			1440 _partID_			1440 _partID_			1440 _partID_		
Observations	8820			8820			8820			8820			8820		
Marginal R^2^/Conditional R^2^	0.000/0.963			0.004/0.963			0.010/0.963			0.022/0.962			0.023/0.962		

Model 1 reports unadjusted estimates; Model 2 has been adjusted for age and sex; Model 3 has been adjusted for age, sex, physical exercise, sleep, and smoking. Model 4 has been adjusted for age, sex, physical exercise, sleep, smoking, religion, location, asset quintiles, and season; Model 5 has been adjusted for age, sex, physical exercise, sleep, smoking, religion, location, asset quintiles, season, hypertension, diabetes, any kidney disease, heart disease, and stroke; σ^2^: Population variance; τ_00_: Random intercept variance; ICC: Intraclass correlation coefficient.

**Table 4:** Association between 24 h urinary sodium and mean waist-to-hip ratio.

	Model 1			Model 2			Model 3			Model 4			Model 5		
Predictors	Estimates	95% CI	*p*-Value	Estimates	95% CI	*p*-Value	Estimates	95% CI	*p*-Value	Estimates	95% CI	*p*-Value	Estimates	95% CI	*p*-Value
Intercept	0.884	0.880–0.888	<0.001	0.927	0.859–0.995	<0.001	0.931	0.863–0.998	<0.001	0.919	0.853–0.986	<0.001	0.918	0.852–0.985	<0.001
Sodium (100 mmol)	0.000	−0.001–0.002	0.67	−0.000	−0.001–0.001	0.91	0.000	−0.001–0.002	0.64	0.001	−0.000–0.002	0.17	0.001	−0.000–0.002	0.14
Age (Years)				0.001	0.001–0.001	<0.001	0.001	0.001–0.001	<0.001	0.001	0.001–0.001	<0.001	0.001	0.001–0.001	<0.001
Sex (Male)				0.040	0.033–0.048	<0.001	0.044	0.037–0.051	<0.001	0.043	0.036–0.050	<0.001	0.043	0.036–0.050	<0.001
Height (cms)				−0.002	−0.003–−0.002	<0.001	−0.002	−0.003–−0.002	<0.001	−0.002	−0.003–−0.002	<0.001	−0.002	−0.003–−0.002	<0.001
Weight (kg)				0.005	0.004–0.005	<0.001	0.005	0.004–0.005	<0.001	0.005	0.004–0.005	<0.001	0.005	0.004–0.005	<0.001
Physical Exercise (Yes)							−0.003	−0.005–−0.002	<0.001	−0.003	−0.004–−0.001	<0.001	−0.003	−0.004–−0.001	<0.001
Sleep (Yes)							−0.001	−0.004–0.001	0.30	−0.001	−0.004–0.001	0.30	−0.001	−0.004–0.001	0.30
Smoker (Yes)							−0.003	−0.005–−0.002	<0.001	−0.002	−0.003–0.000	0.07	−0.002	−0.004–0.000	0.06
Drink Alcohol (Yes)							−0.008	−0.019–0.003	0.14	−0.006	−0.017–0.005	0.26	−0.007	−0.017–0.004	0.21
Religion (Islam)										0.010	0.004–0.016	<0.001	0.010	0.004–0.016	<0.001
Location (Coastal)										−0.019	−0.026–−0.011	<0.001	−0.019	−0.026–−0.011	<0.001
Asset Quintile										0.000	−0.002–0.002	0.98	0.000	−0.002–0.002	0.97
Season (Wet)										0.007	0.004–0.009	<0.001	0.007	0.004–0.009	<0.001
Hypertension													−0.000	−0.000–0.000	0.84
Diabetes													0.000	−0.000–0.000	0.15
Any kidney disease													−0.000	−0.000–0.000	0.10
Any heart disease													−0.001	−0.001–−0.000	<0.001
Stroke													−0.000	−0.001–0.001	0.94
Random Effects															
σ^2^	0.001			0.001			0.001			0.001			0.001		
τ_00_	0.005 _partID_			0.003 _partID_			0.003 _partID_			0.002 _partID_			0.002 _partID_		
ICC	0.801			0.695			0.694			0.685			0.686		
N	1440 _partID_			1440 _partID_			1440 _partID_			1440 _partID_			1440 _partID_		
Observations	8820			8820			8820			8820			8820		
Marginal R^2^/Conditional R^2^	0.000/0.801			0.403/0.818			0.402/0.817			0.419/0.817			0.420/0.818		

Model 1 reports unadjusted estimates; Model 2 has been adjusted for age, sex, height, and weight; Model 3 has been adjusted for age, sex, height, weight, physical exercise, sleep, and smoking; Model 4 has been adjusted for age, sex, height, weight, physical exercise, sleep, smoking, religion, location, asset quintiles, and season; Model 5 has been adjusted for age, sex, height, weight, physical exercise, sleep, smoking, religion, location, asset quintiles, season, hypertension, diabetes, any kidney disease, heart disease, and stroke; σ^2^: Population variance; τ_00_: Random intercept variance; ICC: Intraclass correlation coefficient.

**Table 5:** Association between 24 h urinary sodium and mean waist-to-height ratio.

	Model 1			Model 2			Model 3			Model 4			Model 5		
Predictors	Estimates	95% CI	*p*-Value	Estimates	95% CI	*p*-Value	Estimates	95% CI	*p*-Value	Estimates	95% CI	*p*-Value	Estimates	95% CI	*p*-Value
Intercept	0.507	0.503–0.511	<0.001	0.206	0.194–0.218	<0.001	0.209	0.196–0.221	<0.001	0.224	0.211–0.238	<0.001	0.224	0.211–0.238	<0.001
Sodium (100 mmol)	0.001	0.001–0.002	<0.001	0.001	0.000–0.002	<0.001	0.001	0.000–0.002	<0.001	0.001	0.001–0.002	<0.001	0.001	0.001–0.002	<0.001
Age (Years)				0.001	0.001–0.001	<0.001	0.001	0.001–0.001	<0.001	0.001	0.001–0.001	<0.001	0.001	0.001–0.001	<0.001
Sex (Male)				−0.044	−0.048–−0.040	<0.001	−0.044	−0.048–−0.040	<0.001	−0.044	−0.048–−0.040	<0.001	−0.044	−0.048–−0.040	<0.001
Weight in kg				0.005	0.005–0.005	<0.001	0.005	0.005–0.005	<0.001	0.005	0.005–0.005	<0.001	0.005	0.005–0.005	<0.001
Physical Exercise (Yes)							−0.002	−0.002–−0.001	<0.001	−0.002	−0.002–−0.001	<0.001	−0.002	−0.002–−0.001	<0.001
Sleep (Yes)							−0.000	−0.001–0.001	0.96	−0.000	−0.001–0.001	0.98	−0.000	−0.001–0.001	0.97
Smoker (Yes)							0.000	−0.001–0.001	0.92	0.001	−0.000–0.002	0.09	0.001	−0.000–0.002	0.09
Drink Alcohol (Yes)							−0.002	−0.008–0.005	0.59	−0.001	−0.007–0.005	0.77	−0.001	−0.007–0.005	0.74
Religion (Islam)										0.003	−0.001–0.008	0.14	0.003	−0.001–0.008	0.14
Location (Coastal)										−0.022	−0.027–−0.016	<0.001	−0.022	−0.027–−0.016	<0.001
Asset Quintile										−0.001	−0.002–0.000	0.17	−0.001	−0.002–0.000	0.17
Season (Wet)										0.003	0.002–0.005	<0.001	0.003	0.002–0.005	<0.001
Hypertension													−0.000	−0.000–0.000	0.91
Diabetes													0.000	−0.000–0.000	0.40
Any kidney disease													−0.000	−0.000–0.000	0.29
Any heart disease													−0.000	−0.000–0.000	0.20
Stroke													−0.000	−0.000–0.000	0.87
Random Effects															
σ^2^	0.000			0.000			0.000			0.000			0.000		
τ_00_	0.004 _partID_			0.002 _partID_			0.002 _partID_			0.002 _partID_			0.002 _partID_		
ICC	0.914			0.834			0.834			0.826			0.826		
N	1440 _partID_			1440 _partID_			1440 _partID_			1440 _partID_			1440 _partID_		
Observations	8820			8820			8820			8820			8820		
Marginal R^2^/Conditional R^2^	0.000/0.914			0.590/0.932			0.590/0.932			0.607/0.932			0.607/0.932		

Model 1 reports unadjusted estimates; Model 2 has been adjusted for age, sex, and weight; Model 3 has been adjusted for age, sex, weight, physical exercise, sleep, and smoking; Model 4 has been adjusted for age, sex, weight, physical exercise, sleep, smoking, religion, location, asset quintiles, and season; Model 5 has been adjusted for age, sex, weight, physical exercise, sleep, smoking, religion, location, asset quintiles, season, hypertension, diabetes, any kidney disease, heart disease, and stroke; σ^2^: Population variance; τ_00_: Random intercept variance; ICC: Intraclass correlation coefficient.

**Table 6:** Association between 24 h urinary sodium and mean body fat percentage.

	Model 1			Model 2			Model 3			Model 4			Model 5		
Predictors	Estimates	95% CI	*p*-Value	Estimates	95% CI	*p*-Value	Estimates	95% CI	*p*-Value	Estimates	95% CI	*p*-Value	Estimates	95% CI	*p*-Value
Intercept	28.1	27.4–28.8	<0.001	54.9	48.5–61.3	<0.001	54.9	48.5–61.2	<0.001	55.8	49.3–62.2	<0.001	55.9	49.4–62.3	<0.001
Sodium (100 mmol)	0.3	0.1–0.6	0.01	0.2	−0.1–0.4	0.19	0.2	−0.1–0.4	0.20	0.1	−0.2–0.3	0.54	0.1	−0.2–0.3	0.53
Age (Years)				0.1	0.1–0.1	<0.001	0.1	0.1–0.1	<0.001	0.1	0.1–0.1	<0.001	0.1	0.1–0.1	<0.001
Sex (Male)				−9.0	−9.7–−8.3	<0.001	−8.9	−9.7–−8.2	<0.001	−8.9	−9.6–−8.2	<0.001	−8.8	−9.6–−8.1	<0.001
Height (cms)				−0.4	−0.4–−0.3	<0.001	−0.4	−0.4–−0.3	<0.001	−0.4	−0.4–−0.3	<0.001	−0.4	−0.4–−0.3	<0.001
Weight (kg)				0.5	0.5–0.6	<0.001	0.5	0.5–0.6	<0.001	0.5	0.5–0.6	<0.001	0.5	0.5–0.6	<0.001
Physical Exercise							0.1	−0.1–0.3	0.23	0.0	−0.2–0.2	0.71	0.0	−0.2–0.2	0.75
Sleep							−0.3	−0.6–0.0	0.08	−0.3	−0.7–0.0	0.05	−0.3	−0.7–0.0	0.05
Smoker							−0.3	−0.6–0.1	0.10	−0.3	−0.7–0.0	0.05	−0.3	−0.7–0.0	0.05
Drink Alcohol (Yes)							2.7	1.3–4.2	<0.001	2.4	0.9–3.9	<0.001	2.3	0.8–3.7	<0.001
Religion (Islam)										−0.6	−1.1–−0.0	0.05	−0.6	−1.2–−0.0	0.04
Location (Coastal)										−0.3	−0.8–0.3	0.36	−0.3	−0.8–0.3	0.32
Asset Quintile										−0.1	−0.2–0.1	0.39	−0.1	−0.2–0.1	0.40
Season (Wet)										−0.7	−1.0–−0.5	<0.001	−0.7	−1.0–−0.4	<0.001
Hypertension													−0.0	−0.0–−0.0	0.02
Diabetes													−0.0	−0.0–0.0	0.44
Any kidney disease													0.0	−0.0–0.0	0.31
Any heart disease													0.0	−0.0–0.0	0.87
Stroke													0.0	−0.0–0.1	0.89
Random Effects															
σ^2^	12.13			11.65			11.61			11.44			11.41		
τ_00_	54.39 _partID_			5.53 _partID_			5.42 _partID_			5.52 _partID_			5.59 _partID_		
ICC	0.82			0.32			0.32			0.33			0.33		
N	591 _partID_			591 _partID_			591 _partID_			591 _partID_			591 _partID_		
Observations	2951			2951			2951			2951			2951		
Marginal R^2^/Conditional R^2^	0.001/0.818			0.738/0.822			0.740/0.823			0.741/0.826			0.741/0.826		

Model 1 reports unadjusted estimates; Model 2 has been adjusted for age, sex, and weight; Model 3 has been adjusted for age, sex, weight, physical exercise, sleep, and smoking; Model 4 has been adjusted for age, sex, weight, physical exercise, sleep, smoking, religion, location, asset quintiles, and season; Model 5 has been adjusted for age, sex, weight, physical exercise, sleep, smoking, religion, location, asset quintiles, season, hypertension, diabetes, any kidney disease, heart disease, and stroke; σ^2^: Population variance. τ_00_: Random intercept variance; ICC: Intraclass correlation coefficient.

**Table 7:** Association between 24 h urinary sodium and mean visceral fat percentage.

	Model 1			Model 2			Model 3			Model 4			Model 5		
Predictors	Estimates	95% CI	*p*-Value	Estimates	95% CI	*p*-Value	Estimates	95% CI	*p*-Value	Estimates	95% CI	*p*-Value	Estimates	95% CI	*p*-Value
Intercept	6.5	6.1–6.8	<0.001	26.6	24.1–29.0	<0.001	26.4	24.0–28.8	<0.001	26.1	23.7–28.5	<0.001	26.1	23.7–28.5	<0.001
Sodium (100 mmol)	0.1	−0.0–0.1	0.15	−0.0	−0.1–0.1	0.87	−0.0	−0.1–0.1	0.89	−0.0	−0.1–0.1	0.90	−0.0	−0.1–0.1	0.90
Age (Years)				0.0	0.0–0.0	<0.001	0.0	0.0–0.0	<0.001	0.0	0.0–0.0	<0.001	0.0	0.0–0.0	<0.001
Sex (Male)				2.0	1.8–2.3	<0.001	2.0	1.7–2.3	<0.001	2.0	1.7–2.3	<0.001	2.0	1.7–2.3	<0.001
Height (cms)				−0.3	−0.3–−0.3	<0.001	−0.3	−0.3–−0.3	<0.001	−0.3	−0.3–−0.3	<0.001	−0.3	−0.3–−0.3	<0.001
Weight (kg)				0.4	0.4–0.4	<0.001	0.4	0.4–0.4	<0.001	0.4	0.4–0.4	<0.001	0.4	0.4–0.4	<0.001
Physical Exercise							−0.0	−0.1–0.0	0.39	−0.0	−0.1–0.0	0.30	−0.0	−0.1–0.0	0.32
Sleep							0.1	−0.0–0.2	0.14	0.1	−0.0–0.2	0.15	0.1	−0.0–0.2	0.14
Smoker							0.0	−0.1–0.1	0.46	0.0	−0.1–0.1	0.54	0.0	−0.1–0.1	0.56
Drink Alcohol (Yes)							0.7	0.3–1.1	<0.001	0.6	0.2–1.0	<0.001	0.6	0.2–1.1	<0.001
Religion (Islam)										0.0	−0.2–0.3	0.71	0.0	−0.2–0.3	0.70
Location (Coastal)										−0.2	−0.4–0.0	0.11	−0.2	−0.4–0.0	0.10
Asset Quintile										0.0	−0.0–0.1	0.31	0.0	−0.0–0.1	0.32
Season (Wet)										0.0	−0.1–0.1	1.00	0.0	−0.1–0.1	0.84
Hypertension													0.0	−0.0–0.0	0.96
Diabetes													−0.0	−0.0–0.0	0.53
Any kidney disease													−0.0	−0.0–0.0	0.83
Any heart disease													0.0	−0.0–0.0	0.54
Stroke													−0.0	−0.0–0.0	0.66
Random Effects															
σ^2^	1.04			0.69			0.69			0.69			0.69		
τ_00_	16.72 _partID_			1.02 _partID_			1.00 _partID_			1.00 _partID_			1.00 _partID_		
ICC	0.94			0.60			0.59			0.59			0.59		
N	591 _partID_			591 _partID_			591 _partID_			591 _partID_			591 _partID_		
Observations	2951			2951			2951			2951			2951		
Marginal R^2^/Conditional R^2^	0.000/0.941			0.904/0.961			0.905/0.961			0.905/0.961			0.905/0.961		

Model 1 reports unadjusted estimates; Model 2 has been adjusted for age, sex, and weight; Model 3 has been adjusted for age, sex, weight, physical exercise, sleep, and smoking; Model 4 has been adjusted for age, sex, weight, physical exercise, sleep, smoking, religion, location, asset quintiles, and season; Model 5 has been adjusted for age, sex, weight, physical exercise, sleep, smoking, religion, location, asset quintiles, season, hypertension, diabetes, any kidney disease, heart disease, and stroke; σ^2^: Population variance; τ_00_: Random intercept variance; ICC: Intraclass correlation coefficient.

**Table 8:** Association between 24 h urinary sodium and 90% percentile waist circumference, BMI, waist-to-hip ratio and waist-to-height ratio.

	Waist Circumference in cms			BMI			Waist-to-Hip Ratio			Waist-to-Height Ratio		
Predictors	Estimates	95% CI	*p*-Value	Estimates	95% CI	*p*-Value	Estimates	95% CI	*p*-Value	Estimates	95% CI	*p*-Value
Intercept	79.413	72.977–85.849	<0.001	21.317	20.292–22.342	<0.001	0.996	0.918–1.075	<0.001	0.241	0.224–0.258	<0.001
Sodium (100 mmol)	0.229	0.027–0.432	0.027	0.585	0.388–0.782	<0.001	0.003	0–0.006	0.024	−0.001	−0.003–0.001	0.496
Age (Years)	0.09	0.045–0.136	<0.001	−0.003	−0.025–0.018	0.771	0.002	0.001–0.003	<0.001	0.001	0.001–0.002	<0.001
Sex (Male)	0.498	−0.124–1.119	0.116	−0.394	−0.797–0.009	0.055	0.052	0.044–0.06	<0.001	−0.056	−0.062–0.05	<0.001
Height (cms)	−0.325	−0.372–0.278	<0.001	−	−	−	0.006	0–0.012	0.05	−	−	−
Weight (kg)	0.96	0.893–1.027	<0.001	−	−	−	0.002	0.001–0.004	0.011	0.006	0.005–0.006	<0.001
Physical Exercise	−0.438	−0.652–0.225	<0.001	−0.118	−0.295–0.06	0.193	−0.007	−0.01–0.003	<0.001	−0.005	−0.007–0.002	<0.001
Sleep	0.007	−0.372–0.387	0.969	0.4	0.111–0.69	0.007	−0.003	−0.008–0.002	0.264	0.002	−0.002–0.006	0.261
Smoker	0.181	−0.049–0.411	0.122	−0.349	−0.542–0.156	<0.001	0	−0.003–0.003	0.933	0.002	−0.001–0.004	0.125
Drink Alcohol (Yes)	−0.064	−1.154–1.027	0.909	0.36	−0.794–1.515	0.539	0.003	−0.011–0.018	0.674	0	−0.012–0.012	0.987
Religion (Islam)	0.079	−0.454–0.612	0.77	0.338	−0.129–0.806	0.156	0.009	0.002–0.015	0.009	0.001	−0.004–0.006	0.674
Location (Coastal)	−2.544	−3.154–1.934	<0.001	−0.177	−0.77–0.415	0.556	−0.019	−0.027–0.012	<0.001	−0.024	−0.03–0.018	<0.001
Asset Quintile	−0.022	−0.206–0.163	0.817	0.665	0.498–0.832	<0.001	0	−0.003–0.002	0.826	−0.001	−0.003–0	0.128
Season (Wet)	0.446	0.186–0.707	0.001	0.356	0.179–0.532	<0.001	0.011	0.008–0.014	<0.001	0.002	−0.001–0.005	0.123
Hypertension	0	−0.067–0.067	0.995	−0.004	−0.023–0.015	0.652	0	0–0	0.958	0	0–0	0.279
Diabetes	0.009	−0.027–0.045	0.627	0.001	−0.01–0.011	0.879	0	−0.001–0	0.519	0	0–0	0.793
Any kidney disease	−0.006	−0.045–0.033	0.761	−0.002	−0.011–0.006	0.576	0	0–0	0.761	0	0–0	0.234
Any heart disease	−0.027	−0.202–0.148	0.764	−0.009	−0.292–0.274	0.95	−0.001	−0.004–0.003	0.799	0	−0.002–0.002	0.784
Stroke	−0.026	−0.498–0.446	0.915	−0.018	−0.588–0.553	0.952	0	−0.009–0.009	0.995	0	−0.004–0.004	0.98
Random Effects												
σ^2^	20.68			3.28			0.024			7.41		
τ_00_	4.13 _partID_			4.30 _partID_			1.00 _partID_			1.00 _partID_		
ICC	0.17			0.57			0.97			0.99		
N	1440 _partID_			1440 _partID_			1440 _partID_			1440 _partID_		
Observations	8820			8820			8820			8820		

σ^2^: Population variance; τ_00_: Random intercept variance; ICC: Intraclass correlation coefficient.

**Table 9:** Association between 24 h urinary sodium and 90th percentile body fat percentage and visceral fat percentage metabolism.

	Body Fat %			Visceral Fat %		
Predictors	Estimates	95% CI	*p*-Value	Estimates	95% CI	*p*-Value
Intercept	55.041	47.137–62.945	<0.001	26.225	23.112–29.337	<0.001
Sodium (100 mmol)	0.187	−0.012–0.386	0.065	−0.036	−0.117–0.044	0.374
Age (Years)	0.115	0.098–0.132	<0.001	0.04	0.031–0.049	<0.001
Sex (Male)	−8.862	−9.772–7.952	<0.001	1.804	1.488–2.12	<0.001
Height (cms)	−0.354	−0.406–0.302	<0.001	−0.293	−0.319–0.268	<0.001
Weight (kg)	0.538	0.508–0.567	<0.001	0.442	0.418–0.466	<0.001
Physical Exercise	0.146	−0.099–0.39	0.242	−0.022	−0.105–0.061	0.605
Sleep	−0.278	−0.638–0.082	0.13	0.032	−0.094–0.157	0.617
Smoker	−0.417	−0.934–0.099	0.113	0.265	0.079–0.451	0.005
Drink Alcohol (Yes)	2.223	−4.254–8.7	0.5	1.01	−0.118–2.138	0.079
Religion (Islam)	−0.398	−0.917–0.12	0.132	0	−0.215–0.215	1
Location (Coastal)	−0.125	−0.599–0.349	0.603	−0.173	−0.4–0.054	0.134
Asset Quintile	−0.071	−0.191–0.05	0.25	0.021	−0.028–0.07	0.397
Season (Wet)	−0.641	−0.903–0.378	<0.001	0.01	−0.071–0.091	0.811
Hypertension	−0.009	−0.02–0.001	0.09	0.002	−0.002–0.006	0.427
Diabetes	0.001	−0.004–0.006	0.705	0.001	−0.003–0.005	0.574
Any kidney disease	0.002	−0.002–0.005	0.426	0	−0.003–0.003	0.978
Any heart disease	0.011	−0.197–0.218	0.919	0.001	−0.061–0.063	0.974
Stroke	0.012	−0.471–0.495	0.962	−0.008	−0.137–0.122	0.907
Random Effects						
σ^2^	13.1			1.34		
τ_00_	1.0 _partID_			1.0 _partID_		
ICC	0.07			0.43		
N	591 _partID_			591 _partID_		
Observations	2951			2951		

σ^2^: Population variance; τ_00_: Random intercept variance; ICC: Intraclass correlation coefficient.

**Table 10:** Association between 24 h urinary sodium and 10th percentile waist circumference, BMI, waist-to-hip ratio and waist-to-height ratio.

	Waist Circumference in cms			BMI			Waist-to-Hip Ratio			Waist-to-Height Ratio		
Predictors	Estimates	95% CI	*p*-Value	Estimates	CI	*p*-Value	Estimates	95% CI	*p*-Value	Estimates	95% CI	*p*-Value
(Intercept)	79.403	72.967–85.838	<0.001	21.194	20.167–22.222	<0.001	0.996	0.917–1.074	<0.001	0.241	0.224–0.258	0<0.001
Sodium (100 mmol)	0.181	−0.033–0.396	0.098	0.385	0.229–0.541	<0.001	0.003	0–0.005	0.051	−0.001	−0.003–0.001	0.293
Age (Years)	0.09	0.063–0.118	<0.001	−0.04	−0.054-−0.026	<0.001	0.001	0.001–0.001	<0.001	0.001	0.001–0.002	<0.001
Sex (Male)	0.59	−0.039–1.219	0.066	−0.35	−0.738–0.038	0.077	0.053	0.045–0.061	<0.001	−0.056	−0.062-−0.05	<0.001
Height (cms)	−0.369	−0.42-−0.319	<0.001	-	-	-	−0.003	−0.005-−0.001	0.009	-	-	-
Weight (kg)	0.967	0.905–1.028	<0.001	-	-	-	0.004	0.004–0.005	<0.001	0.004	0.005–0.006	<0.001
Physical Exercise	−0.41	−0.624-−0.195	<0.001	−0.148	−0.32–0.023	0.09	−0.006	−0.009-−0.003	<0.001	−0.005	−0.007-−0.002	<0.001
Sleep	0.013	−0.366–0.391	0.947	0.295	0.011–0.579	0.042	−0.003	−0.008–0.002	0.206	0.002	−0.002–0.006	0.368
Smoker	0.19	−0.036–0.416	0.1	−0.343	−0.522-−0.164	<0.001	0.001	−0.002–0.004	0.686	0.002	−0.001–0.004	0.124
Drink Alcohol (Yes)	−0.056	−1.147–1.034	0.919	0.351	−0.805–1.506	0.551	0.003	−0.011–0.018	0.667	0	−0.012–0.012	0.987
Religion (Islam)	0.102	−0.431–0.635	0.706	0.202	−0.245–0.65	0.374	0.008	0.002–0.015	0.012	0.001	−0.004–0.006	0.681
Location (Coastal)	−2.63	−3.235-−2.025	<0.001	−0.187	−0.767–0.393	0.525	−0.02	−0.027-−0.012	<0.001	−0.024	−0.03-−0.018	<0.001
Asset Quintile	−0.076	−0.236–0.085	0.355	0.332	0.199–0.466	<0.001	−0.001	−0.003–0.002	0.591	−0.002	−0.003–0	0.049
Season (Wet)	0.471	0.211–0.731	<0.001	0.295	0.118–0.472	0.001	0.011	0.008–0.014	<0.001	0.002	−0.001–0.005	0.13
Hypertension	−0.001	−0.057–0.056	0.975	−0.002	−0.013–0.009	0.709	0	0–0	0.402	0	0–0	0.429
Diabetes	0.006	−0.025–0.036	0.712	−0.003	−0.009–0.002	0.264	0	0–0	0.251	0	0–0	0.676
Any kidney disease	−0.005	−0.023–0.013	0.57	−0.001	−0.006–0.004	0.696	0	0–0	0.739	0	0–0	0.668
Any heart disease	−0.024	−0.194–0.146	0.785	0.006	−0.278–0.29	0.965	0	−0.004–0.004	0.815	0	−0.002–0.002	0.961
Stroke	−0.006	−0.485–0.473	0.981	0.018	−0.55–0.585	0.952	0	−0.009–0.009	0.982	0	−0.004–0.004	0.972
Random Effects												
σ^2^	17.21			2.24			0.01			0.005		
τ_00_	3.52 _partID_			3.18 _partID_			1.00 _partID_			1.00 _partID_		
ICC	0.17			0.59			0.99			0.99		
N	1440 _partID_			1440 _partID_			1440 _partID_			1440 _partID_		
Observations	8820			8820			8820			8820		

σ^2^: Population variance; τ_00_: Random intercept variance; ICC: Intraclass correlation coefficient.

**Table 11:** Association between 24 h urinary sodium and 10th percentile body fat percentage and visceral fat percentage.

	Body Fat %			Visceral Fat %		
Predictors	Estimates	95% CI	*p*-Value	Estimates	95% CI	*p*-Value
(Intercept)	55.041	47.137−62.945	<0.001	26.225	23.112−29.337	<0.001
Sodium (100 mmol)	0.19	−0.009−0.389	0.061	−0.036	−0.117−0.044	0.373
Age (Years)	0.093	0.065−0.121	<0.001	0.036	0.03−0.042	<0.001
Sex (Male)	−8.868	−9.779-−7.956	<0.001	1.804	1.488−2.12	<0.001
Height (cms)	−0.381	−0.435-−0.326	<0.001	−0.3	−0.325-−0.274	<0.001
Weight (kg)	0.557	0.523−0.591	<0.001	0.428	0.409−0.446	<0.001
Physical Exercise	0.149	−0.094−0.393	0.228	−0.021	−0.105−0.062	0.617
Sleep	−0.282	−0.644−0.079	0.125	0.032	−0.093−0.157	0.616
Smoker	−0.427	−0.942−0.088	0.104	0.265	0.079−0.451	0.005
Drink Alcohol (Yes)	2.218	−4.255−8.691	0.5	1.01	−0.118−2.138	0.079
Religion (Islam)	−0.396	−0.913−0.122	0.134	0	−0.215−0.215	0.999
Location (Coastal)	−0.128	−0.603−0.346	0.595	−0.173	−0.4−0.054	0.134
Asset Quintile	−0.068	−0.191−0.055	0.278	0.02	−0.029−0.068	0.421
Season (Wet)	−0.647	−0.909-−0.385	<0.001	0.009	−0.072−0.09	0.829
Hypertension	−0.007	−0.039−0.025	0.667	0.001	−0.003−0.005	0.489
Diabetes	−0.008	−0.024−0.008	0.319	−0.001	−0.004−0.002	0.584
Any kidney disease	0.003	−0.007−0.013	0.562	−0.001	−0.003−0.002	0.575
Any heart disease	0.013	−0.196−0.222	0.902	0	−0.062−0.061	0.993
Stroke	0.013	−0.471−0.497	0.959	−0.007	−0.137−0.122	0.913
Random Effects						
σ^2^	19.0			1.16		
τ_00_	1.34 _partID_			1.0 _partID_		
ICC	0.07			0.54		
N	591 _partID_			591 _partID_		
Observations	2951			2951		

σ^2^: Population variance; τ_00_: Random intercept variance; ICC: Intraclass correlation coefficient.

**Table 12:** Association between 24 h urinary sodium and mean waist circumference, BMI, waist-to-hip ratio, and waist-to-height ratio among complete 24 h urine samples based on criteria creatinine index >0.7.

	Waist Circumference in cms			BMI			Waist-to-Hip Ratio			Waist-to-Height Ratio		
Predictors	Estimates	95% CI	*p*-Value	Estimates	95% CI	*p*-Value	Estimates	95% CI	*p*-Value	Estimates	95% CI	*p*-Value
(Intercept)	77.001	71.690–82.312	<0.001	22.400	21.581–23.220	<0.001	0.928	0.858–0.998	<0.001	0.229	0.215–0.243	<0.001
Sodium (100 mmol)	0.226	0.100–0.353	<0.001	0.099	0.065–0.133	<0.001	0.002	0.000–0.003	0.02	0.001	0.001–0.002	<0.001
Age (Years)	0.092	0.077–0.107	<0.001	−0.025	−0.036–−0.014	<0.001	0.001	0.001–0.001	<0.001	0.001	0.001–0.001	<0.001
Sex (Male)	0.201	−0.370–0.773	0.49	−0.040	−0.298–0.218	0.76	0.044	0.036–0.051	<0.001	−0.045	−0.049–−0.041	<0.001
Height (cms)	−0.348	−0.384–−0.312	<0.001				−0.002	−0.003–−0.002	<0.001			
Weight (kg)	0.987	0.964–1.009	<0.001				0.004	0.004–0.005	<0.001	0.005	0.005–0.005	<0.001
Physical Exercise	−0.202	−0.345–−0.059	0.01	−0.058	−0.099–−0.017	0.01	−0.003	−0.005–−0.001	<0.001	−0.002	−0.003–−0.001	<0.001
Sleep	0.193	−0.045–0.430	0.11	−0.009	−0.081–0.063	0.81	−0.000	−0.003–0.003	0.92	0.001	−0.000–0.003	0.15
Smoker	0.252	0.091–0.412	<0.001	−0.171	−0.221–−0.121	<0.001	−0.001	−0.003–0.001	0.41	0.001	−0.000–0.002	0.10
Drink Alcohol (Yes)	−0.345	−1.292–0.601	0.47	0.237	−0.087–0.562	0.15	−0.008	−0.020–0.004	0.22	−0.002	−0.009–0.006	0.66
Religion (Islam)	0.308	−0.141–0.757	0.18	0.364	−0.044–0.772	0.08	0.010	0.004–0.016	<0.001	0.003	−0.001–0.008	0.17
Location (Coastal)	−2.203	−2.804–−1.603	<0.001	−0.118	−0.645–0.410	0.66	−0.018	−0.026–−0.010	<0.001	−0.020	−0.026–−0.015	<0.001
Asset Quintile	0.015	−0.130–0.160	0.84	0.255	0.153–0.358	<0.001	0.000	−0.002–0.002	0.76	−0.001	−0.002–0.000	0.15
Season (Wet)	0.420	0.172–0.669	<0.001	0.061	−0.004–0.126	0.06	0.008	0.005–0.011	<0.001	0.002	0.001–0.004	<0.001
Hypertension	−0.000	−0.017–0.017	0.97	−0.003	−0.007–0.002	0.24	−0.000	−0.000–0.000	0.88	−0.000	−0.000–0.000	0.88
Diabetes	0.005	−0.008–0.017	0.46	0.001	−0.002–0.004	0.59	0.000	−0.000–0.000	0.45	0.000	−0.000–0.000	0.65
Any kidney disease	−0.006	−0.017–0.005	0.28	0.000	−0.003–0.003	0.98	−0.000	−0.000–0.000	0.31	−0.000	−0.000–0.000	0.45
Any heart disease	−0.014	−0.036–0.008	0.20	0.003	−0.003–0.008	0.35	−0.001	−0.001–−0.000	<0.001	−0.000	−0.000–0.000	0.19
Stroke	−0.002	−0.047–0.042	0.92	0.001	−0.011–0.013	0.87	−0.000	−0.001–0.001	0.99	0.000	−0.000–0.000	0.99
Random Effects												
σ^2^	7.856			0.515			0.001			0.000		
τ_00_	13.589 _partID_			12.832 _partID_			0.002 _partID_			0.001 _partID_		
ICC	0.634			0.961			0.678			0.814		
N	1402 _partID_			1402 _partID_			1402 _partID_			1402 _partID_		
Observations	7221			7221			7221			7221		
Marginal R^2^/Conditional R^2^	0.796/0.925			0.027/0.962			0.398/0.806			0.584/0.923		

σ^2^: Population variance; τ_00_: Random intercept variance; ICC: Intraclass correlation coefficient.

## Data Availability

Data for this manuscript can be obtained by contacting the corresponding author with a reasonable request.

## Supplementary Materials

The following supporting information can be downloaded at: https://www.mdpi.com/article/10.3390/nu14143000/s1, Figure S1: Cubic spline plots of 24-h urinary sodium excretion and waist circumference, BMI, waist to hip ratio and waist to height ratio, Figure S2: Cubic spline plots of 24-h urinary sodium excretion and body fat percentage and visceral fat percentage.
