# Supplementary material for: Urinary Sodium Excretion and Obesity Markers among Bangladeshi Adult Population: Pooled Data from Three Cohort Studies
Source: Nutrients. 2022 Jul 21;14(14):3000. doi: 10.3390/nu14143000 (PMC9323227; doi:10.3390/nu14143000)

Urinary sodium excretion and obesity markers among Bangladeshi adult population: pooled data from three cohort studies

Musarrat J. Rahman[1], Sarker M Parvez[2], Mahbubur Rahman[2], Feng J. He[3], Solveig Cunningham[4], K.M. Venkat Narayan[4], Jaynal Abedin[5], Abu Mohd Naser[6]

[1]International Health, Johns Hopkins Bloomberg School of Public Health, Baltimore, Maryland, USA
[2]Environmental Interventions Unit, Infectious Disease Division, icddr,b, Dhaka, Bangladesh
[3]Centre for Environmental and Preventive Medicine, Wolfson Institute of Preventive Medicine, Barts and The London School of Medicine and Dentistry, Queen Mary University of London, United Kingdom
[4]Emory Global Diabetes Research Center, Hubert Department of Global Health, Rollins School of Public Health, Emory University, Atlanta, GA
[5]Data Science Institute, National University of Ireland Galway, Galway, Ireland
[6]Division of Epidemiology, Biostatistics and Environmental Health, School of Public Health, University of Memphis, Memphis, TN

Correspondence: Dr. Abu Mohd Naser, MBBS, PhD
Email: atitu@memphis.edu
Division of Epidemiology, Biostatistics, and Environmental Health
School of Public Health
University of Memphis

Figure S1: Cubic spline plots of 24-hour urinary sodium excretion and waist circumference, BMI, waist to hip ratio and waist to height ratio

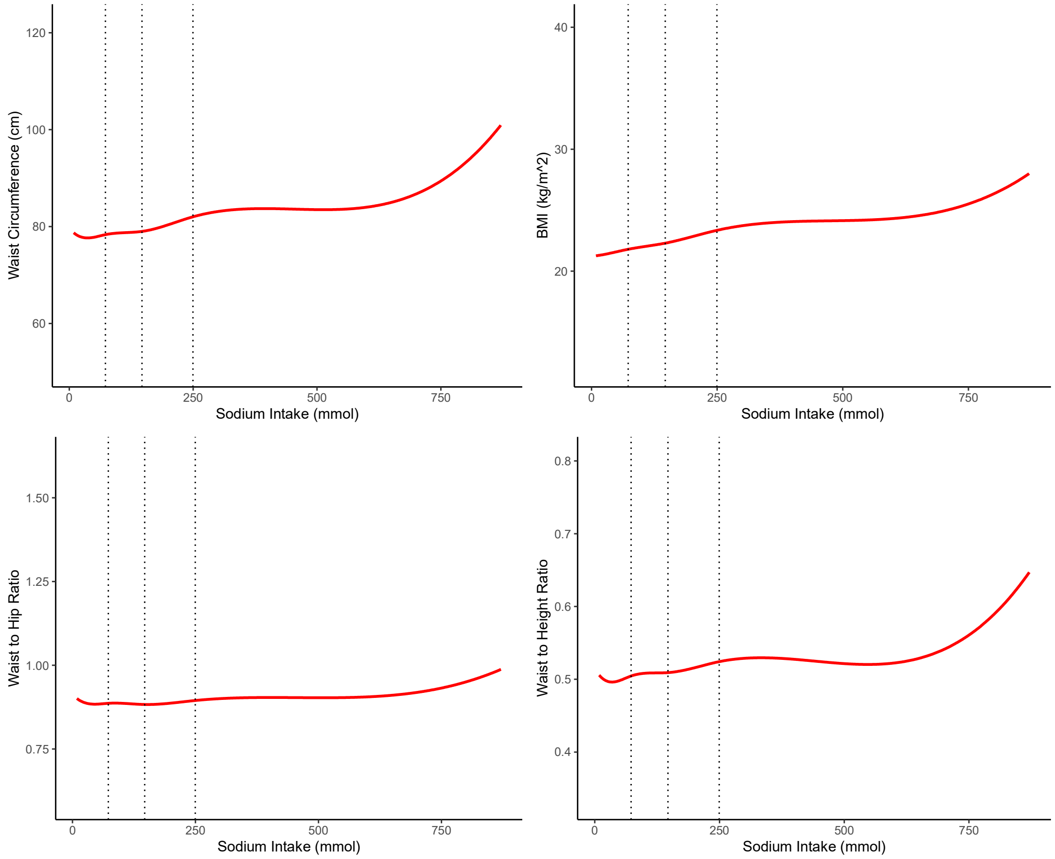

Figure S2: Cubic spline plots of 24-hour urinary sodium excretion and body fat percentage and visceral fat percentage

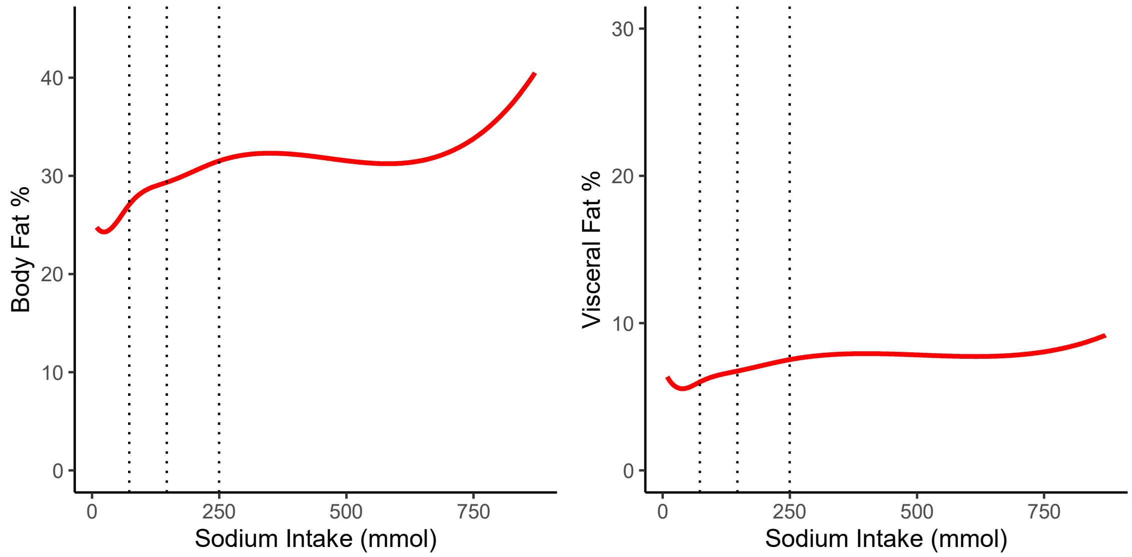

Supplement: Supplementary file 1 [file nutrients-14-03000-s001.zip › nutrients-1777541-supplementary.pdf]
